# Can immigrants counteract employer discrimination? A factorial field experiment reveals the immutability of ethnic hierarchies

**DOI:** 10.1371/journal.pone.0218044

**Published:** 2019-07-24

**Authors:** Kåre Vernby, Rafaela Dancygier

**Affiliations:** 1 Department of Political Science, Stockholm University, Stockholm, Sweden; 2 Department of Politics and Woodrow Wilson School of Public and International Affairs, Princeton University, Princeton, New Jersey, United States of America; TED University, TURKEY

## Abstract

How pervasive is labor market discrimination against immigrants and what options do policymakers and migrants have to reduce it? To answer these questions, we conducted a field experiment on employer discrimination in Sweden. Going beyond existing work, we test for a large range of applicant characteristics using a factorial design. We examine whether migrants can affect their employment chances—by adopting citizenship, acquiring work experience, or signaling religious practice—or whether fixed traits such as country of birth or gender are more consequential. We find little systematic evidence that immigrants can do much to reduce discrimination. Rather, ethnic hierarchies are critical: callback rates decline precipitously with the degree of ethno-cultural distance, leaving Iraqis and Somalis, especially if they are male, with much reduced employment chances. These findings highlight that immigrants have few tools at their disposal to escape ethnic penalties and that efforts to reduce discrimination must address employer prejudice.

## Introduction

The economic integration of immigrants is one of the most pressing policy issues facing Europe today. Though many Western European countries have experienced large-scale migration for decades, their records of integrating migrants into domestic labor markets remain patchy. Millions of immigrants and their descendants remain unemployed or underpaid [1]. As a result, a growing literature has begun to investigate barriers to immigrants’ economic success, with one strand focusing on the hurdles that migrants encounter during the hiring process. This research has consistently found that job applicants with immigrant origins are less likely to be invited to interviews than are natives [2].

Given that discrimination is pervasive, what options do policymakers and migrants have at their disposal to escape ethnic penalties? Existing research has demonstrated that foreign origin presents an obstacle in the recruitment process, but it has less frequently considered whether foreign origin is a proxy for other factors that migrants and policy can influence. If discrimination is largely driven by comparatively fixed ascriptive traits such as ethnicity or gender, states should design policies to help break down employer stereotypes. If, by contrast, employers respond to attributes over which immigrants have some control (cf., [3]), such as work experience, citizenship, or signals of religiosity, different policy prescriptions emerge.

To provide answers to these questions, we carried out a correspondence study in Sweden and sent applications from fictitious immigrants and natives to restaurant and café jobs. Our outcome of interest is whether applicants received a callback for an interview. Going beyond much existing work, our study employs a factorial design. This design allows us to efficiently test for the impact of a larger range of characteristics (ascriptive traits, changeable attributes, as well as their combinations) than has been the case in previous work (see Appendix B in S1 File). Importantly, this approach permits us to test whether origin-based penalties diminish or persist once we vary attributes that employers could systematically link to specific countries of origin.

To investigate whether ethnic hierarchies are at work, we consider four origin countries—Sweden, Poland, Iraq or Somalia—that have been shown to occupy different rungs on the ethnic hierarchy ladder. Research on social distance has demonstrated hierarchical orderings, whereby West Europeans and North Americans are positively stereotyped, followed by Southern and Eastern Europeans, Asians and Africans. Such hierarchies have been found to influence views about what groups are preferred neighbors or marriage partners [4–6]. We test whether they also extend to labor markets. Additionally, we test whether another comparatively fixed trait—gender—interacts with different ethnicities to shape hiring decisions.

Since immigrants cannot easily change perceptions of ethnic hierarchies (or their gender), we next turn to attributes that immigrants can to some degree affect: the adoption of citizenship, the accumulation of skills, and the salience of their religious practice. Scholars have documented that immigrant citizens are more likely to be employed and to earn higher wages than are those who have not naturalized [7–9]. One policy prescription for immigrants who want to improve their labor market outcomes is therefore to naturalize. Yet, much of the existing work on the citizenship-employment link is observational and cross-sectional. As a result, it is difficult to disentangle whether citizenship acquisition increases earnings or employment options, or whether unobservable characteristics—such as motivation to work or to stay in the country—cause both naturalization and better economic outcomes. We therefore vary immigrant citizenship and examine whether employers are more likely to call back immigrants who have acquired citizenship.

Another way in which immigrants can potentially boost their employment chances is by accumulating work experience early on. Our study consequently tests whether immigrants are more likely to receive callbacks from employers if they indicate more relevant prior work experience.

Finally, Adida and colleagues [10] find that employers are especially likely to discriminate against Muslim immigrants. One mechanism driving this result are concerns that daily religious practice reduces productivity. Accordingly, some applications prime religious activity, allowing us to examine whether migrants who signal that they are religiously active receive fewer callbacks than those who do not.

Our main findings are as follows. First, employer behavior follows ethnic hierarchies: Native Swedes receive the highest response rate (21%), followed by those born in Poland (17%), Iraq (10%), and lastly Somalia (5%). Callback rates for native Swedes are thus four times higher than they are for applicants born in Somalia.

Moreover, gender matters. Across groups, women receive higher callback rates, leading to staggering gaps in recruitment: The callback rate for Somali men is *ten* times smaller than that for Swedish or Polish women. Groups that feature prominently in the debate about integration problems—young, low-skilled, male immigrants from Muslim-majority countries—encounter the most discrimination, even when their applications are otherwise identical to those of natives. As we discuss below, among Muslim applicants the positive effect of female gender could in part be driven by higher payoffs to integration signals among women when compared to men.

Second, the point estimates for the effects of signaling citizenship, work experience and religious practice are much smaller than those for fixed traits, and the confidence intervals surrounding these effects are large. In other words, there is little precise evidence that characteristics over which immigrants have some control influence hiring decisions and, in turn, that these characteristics generate hierarchically-ordered ethnic penalties. In contrast to arguments about the economic returns to citizenship, we find that employers do not prefer immigrant citizens over non-citizens. Furthermore, effects of work experience are small and imprecisely estimated, implying that increased experience will not reduce immigrant-native employment inequalities in substantively meaningful terms. Finally, we do not find evidence of a penalty tied to priming religious activity. If anything, signals of religious practice slightly raise callback rates, but overall ethnicity trumps these signals.

Our paper advances existing scholarship in several ways. First, by selecting several immigrant groups we can establish the significance of deep-seated ethnic hierarchies and, by implication, the severity of ethnic penalties. While most existing research does not examine such hierarchies (see Appendix B and Table F in S1 File), the few studies that do find mixed results [11–13]. This inconclusiveness may be due to employers differing in the unobserved heuristics they use: some employers might associate an immigrant group with citizenship or work experience, while others do not. Unlike most work, our factorial design explicitly tests whether a range of frequently unobserved non-ethnic characteristics produce observed ethnic penalties.

Second, employing the factorial design in the context of a correspondence study is novel. While this design has been used in consumer research [14] and, more recently, in opinion surveys about immigrants [15], correspondence testers have not realized the methodology’s full potential (see Appendix B in S1 File). In particular, using factorial designs one can simultaneously study the effects of several factors using the same sample size that would be required for the study of one independent variable [16]. Substantively, this design also allows us to examine whether ethnic hierarchies persist when country of birth is interacted with several important background characteristics. Though, for a given sample size, there is some loss in statistical power when estimating interactive (vs. average) treatment effects, the factorial design can test whether applicant attributes intersect to jointly produce discrimination [16].

Third, we are among the first to examine whether citizenship improves the employment chances of immigrants within the context of a correspondence study. Our results indicate that in the Swedish context, naturalization should not necessarily be considered a tool for economic integration. If citizenship improves employment outcomes at all, it is unlikely to do so by influencing employer behavior.

Finally, the fact that ascriptive characteristics like ethnic origin and gender critically impact employment outcomes, and that these fixed traits have much larger effects than do attributes that policy and migrant behavior can actually influence, has significant policy implications. Specifically, efforts that seek to improve immigrants’ economic integration will fall short if they are only targeted at immigrants. Instead, our results suggest that policies should focus at least as much on breaking down prejudice among natives. Additionally, governments should consider measures that level the playing field, such as anonymous job applications [17–19].

## Results

### Registry data results

To provide some background on the immigrant-native employment gap and to illustrate the inferential problems inherent in trying to estimate discrimination using observational data, we draw on government registers. These registry data cover the entire Swedish population and contain information on a range of individual characteristics, such as employment status, income, country of birth, citizenship acquisition, gender, education, age and residential location. They allow us to analyze the employment situation of close to six million Swedish residents.

Like in many European countries, Sweden’s immigrant-origin population consists of a mix of labor migrants, family migrants, and refugees from both within and outside of the European Union (EU). At the end of 2016 the ten countries with the largest stock of migrants were (in descending order): Finland, Syria, Iraq, Poland, Iran, the former Yugoslavia, Somalia, Bosnia and Herzegovina, Germany, and Turkey. Though employment rates vary across groups, the overall economic performance of migrants falls well below that of natives. When compared to other OECD countries, Sweden features the highest gap in employment rates (17.6 percentage points) between immigrants and natives [20].

Table 1 reports group-wise regressions and average employment rates. We observe an ordering consistent with ethnic hierarchies: native Swedes have the highest employment rates (86%), followed by those born in Poland (73%), Iraq (60%), and finally those born in the Horn of Africa (58%) (Somalia is one of the five countries grouped into this latter regional category; for details, see our discussion in the Materials and methods section). Furthermore, coefficient estimates differ across groups. The estimates for educational attainment are positive across the board, but the size is generally larger for Swedes, suggesting that natives reap higher returns from education.

**Table 1:** Correlates of having employment by country of birth.

	(1)	(2)	(3)	(4)	(5)
	Sweden	Outside Sweden	Poland	Iraq	Horn of Africa
Mean outcome	0.8643	0.6908	0.7251	0.5957	0.5795
Gender (Female)	0.0018*** (0.0003)	-0.0420*** (0.0008)	-0.0280*** (0.0038)	-0.1010*** (0.0028)	-0.0885*** (0.0034)
*Education*:					
Elementary school	0.1007*** (0.0031)	0.0603*** (0.0020)	0.0118 (0.0144)	0.0386*** (0.0053)	0.0669*** (0.0059)
Upper secondary school	0.2685*** (0.0030)	0.1944*** (0.0016)	0.1862*** (0.0125)	0.1752*** (0.0045)	0.2109*** (0.0046)
Post-secondary (≤2 years)	0.2801*** (0.0031)	0.1922*** (0.0023)	0.1702*** (0.0144)	0.1641*** (0.0075)	0.2221*** (0.0089)
Post-secondary (≥2 years)	0.3242*** (0.0030)	0.2445*** (0.0016)	0.2234*** (0.0125)	0.2377*** (0.0044)	0.2515*** (0.0059)
Graduate School (PhD)	0.3486*** (0.0032)	0.3274*** (0.0030)	0.2847*** (0.0180)	0.3296*** (0.0178)	0.2011*** (0.0254)
Age	0.0146*** (0.0001)	0.0167*** (0.0002)	0.0226*** (0.0011)	0.0110*** (0.0008)	0.0074*** (0.0010)
Age^2^	-0.0002*** (0.0000)	-0.0003*** (0.0000)	-0.0003*** (0.0000)	-0.0003*** (0.0000)	-0.0002*** (0.0000)
Citizen		0.0698*** (0.0011)	0.1257*** (0.0049)	0.0680*** (0.0050)	0.1183*** (0.0050)
Time in Sweden		0.0139*** (0.0001)	0.0027*** (0.0007)	0.0309*** (0.0008)	0.0273*** (0.0008)
Time in Sweden^2^		-0.0002*** (0.0000)	-0.0001*** (0.0000)	-0.0006*** (0.0000)	-0.0005*** (0.0000)
Constant	0.3698*** (0.0033)	0.1521*** (0.0049)	0.1368*** (0.0254)	0.1715*** (0.0157)	0.2161*** (0.0186)
Observations Adj. R-squared	4,755,922 0.0705	1,112,526 0.0982	55,597 0.0647	105,707 0.131	70,587 0.177

Notes: The dependent variable is a dummy indicating whether an individual had any wage income in 2015 (1) or not (0). OLS coefficients; robust standard errors in parentheses. Significance levels:

*** p<0.01,

** p<0.05,

* p<0.1

Table 1 also includes variables specific to foreign-born groups, of which Time in Sweden has a positive, if decreasing, relationship with employment, with impacts substantively larger for those born in Iraq and the Horn of Africa. Most interestingly, citizenship is strongly correlated with employment for all foreign-born groups, raising the employment probability by between 7 and 13 percentage points.

These results establish a set of important empirical regularities. First, employment gaps are consistent with an ethnic hierarchy, whereby those born in the Horn of Africa face the steepest penalty, followed by individuals born in Iraq, and then Poland. Second, citizenship is positively associated with employment, and it appears to narrow immigrant-native employment gaps.

Yet, these analyses also face shortcomings. First, it is possible that we did not capture variables that lead to both employment gaps between immigrants and natives and to the specific ethnic hierarchies (e.g., group-level variation in confidence, networks, search intensity or choice of sector). Second, the citizenship effect is difficult to interpret. In line with models of statistical discrimination [21–23], citizenship may reduce employer discrimination by signaling commitment to stay in the country or by lessening administrative or legal hurdles that can arise in the hiring of non-citizens [24]. At the same time, citizenship itself might not contribute to employment, but the factors that lead to citizenship acquisition also lead to improved economic outcomes. Immigrants who are motivated to assimilate, to put down roots, or to move up economically might be more likely to acquire citizenship and to exhibit favorable employment outcomes.

Third, it is challenging to measure the effects of prior work experience and immigrant status using observational data. If being born abroad reduces the odds of acquiring work experience due to employer discrimination, the inclusion of prior work experience would lead to a biased estimate (post-treatment bias) of the effect of having been born in a foreign country on employment. We face the same problem with some of the variables that we do include (such as citizenship acquisition and educational attainment), making it difficult to assess their impact; the impact of country of birth generally; and of ethnic hierarchies associated with the countries in our study specifically.

In short, though the analyses relying on registry data provide us with a good overview of employment inequalities, they are not well suited for isolating the causal role that specific individual characteristics play when employers evaluate applicants. We therefore next turn to our correspondence study.

### CV experiment results

Our field experiment is designed to estimate the impact of two types of factors: relatively fixed attributes that individuals cannot readily modify and more malleable features over which individuals have some control and which employers may associate with ethnic backgrounds. Identifying which type of factor generates employer discrimination is critical if we want to make headway in improving immigrants’ labor market integration. While modern racism or prejudice is typically subtle [25], testing studies in hiring processes offer a way to measure discrimination and have been conducted in a growing number of countries to unveil discrimination against different groups [26, 27].

We sent fictitious applications to hiring companies located in Sweden’s seven largest metropolitan regions, which are home to about a third of the country’s immigrant population. We varied applicants’ country of birth, gender, citizenship, amount of previous work experience, and signals of religious activity (we also vary the name, selecting first and last names common in the origin country).

All applications are sent by fictitious immigrants who came to Sweden as children and who were born in 1995 (making them 21 or 22 years of age; the experiment ran from February 2016 to May 2017). We chose this group to minimize presumed immigrant-native differences in unobserved characteristics such as language skills or the quality of education. Moreover, the future of immigrant integration hinges on the younger generation, making this group substantively important. Note that this choice (vs. selecting migrants who arrived as adults) likely reduces the amount of discrimination we observe.

Applications were sent to jobs in the restaurant and café sector (one randomly selected application per job opening). We hold the sector constant, but jobs differ slightly in that some require prior experience (e.g., cashier or waiter) while others do not. All applications mention work at a café during high school, but for jobs that made previous work experience a requirement, we only sent applications that additionally included relevant post-high school work experience. We control for this difference in the analysis.

We selected the restaurant and café sector because, first, it is the sector that currently employs the plurality of immigrants [28]. Second, this sector employs relatively young workers and is an important entry point into the labor market [28]. Third, it employs the highest number of undocumented migrants [29]; if citizenship matters by reducing concerns about hiring undocumented workers, we should observe effects in this sector.

With respect to generalizability, several aspects should be considered. On the one hand, a large immigrant workforce is present in this sector, so our findings could present a lower bound of discrimination. On the other hand, because the jobs involve face-to-face interactions, employers might be concerned about customer prejudice, thereby raising levels of discrimination. We thus expect that our results should best generalize to service jobs that already employ migrant workers, require relatively few skills and involve face-to-face contact with customers. Turning to individual-level attributes, and as we detail in the design section below, to ensure that applications were not rejected across the board we developed cover letters that raised the baseline attractiveness of all applicants. This included, among other attributes, membership in the local football team. Given stereotypes about Muslim women’s engagement with non-Muslims, it is possible that this membership is a signal of integration among female applicants born in Iraq and Somalia. Another feature to be kept in mind when considering generalizability relates to the assignment of treatments in the factorial design. These assignments are based on assessments about statistical power, rather than national representativeness (e.g., exactly half of the immigrants in our design are Swedish citizens/female, but the true proportions in the population are somewhat lower).

The outcome of interest is the callback rate. A job offer, an invitation for an interview, or an inquiry asking about the applicant’s continued interest were coded as 1. Non-responses and all other responses were coded as zero. We first display the callback rates by the ascriptive characteristics that we manipulate: country of birth and gender.

**Fig 1:**
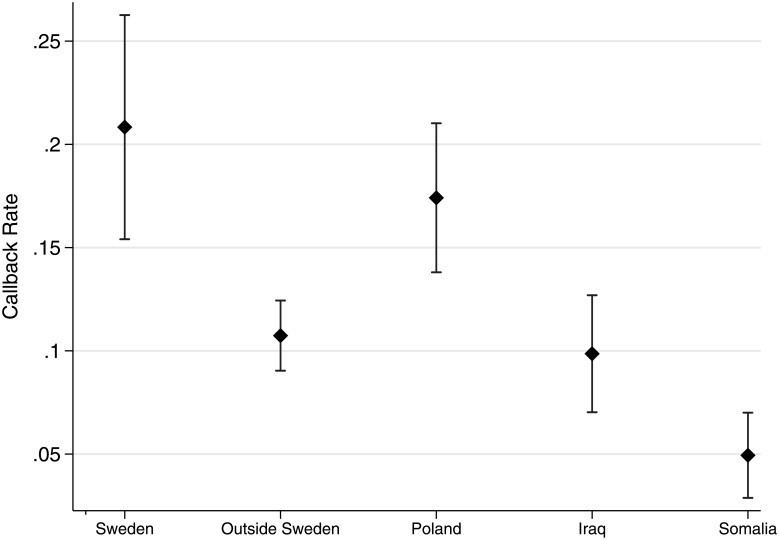
Callback rates by country of birth. Note: Capped bars represent 95% confidence intervals.

Fig 1 demonstrates that ethnic hierarchies indeed govern employer responses. Callback rates are higher for native Swedes (21%) than for applicants born outside of Sweden (11%). And when we disaggregate the foreign-born, it is clear that callback rates differ dramatically across countries of birth; native Swedes are followed by applicants born in Poland (17%), Iraq (10%), and Somalia (5%), respectively. The difference in callback rates between the Polish-born and the Iraqi and Somalia-born are thus substantively large and statistically significant (*p*<0.01), as is the difference between immigrants born in Iraq vs. Somalia (*p*<.01). These experimental results are in line with our observational findings, suggesting that the ethnic ordering of employment rates can at least in part be attributed to employer behavior, rather than simply to unobservable characteristics on which these groups vary.

**Fig 2:**
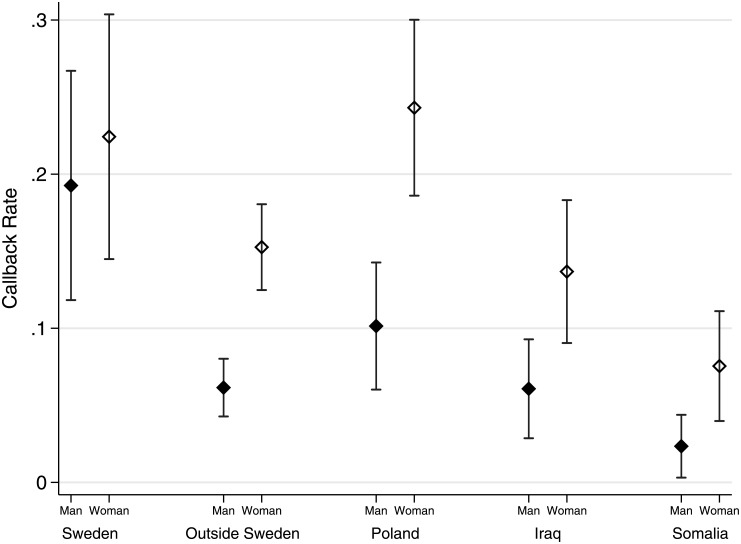
Callback rates by country of birth and gender. Note: Capped bars represent 95% confidence intervals.

Furthermore, Fig 2 demonstrates that callback rates differ dramatically when we take into account both country of birth and gender. Whereas only 2.5% of male Somali-born applicants receive a callback, the corresponding figure for female Somali-born applicants is 7.5% (*p*<0.05). Female applicants receive higher callback rates, and this tendency is especially pronounced among immigrant groups. The lower employment rates among women born in Iraq and Somalia that were apparent in the registry data are therefore unlikely only the result of employer discrimination and instead partly reflect other differences in the drivers of female employment across groups.

The results thus far establish the striking importance of ascriptive traits. Can immigrants escape the penalties imposed by their country of birth and gender? To answer this question, we first examine whether citizenship influences hiring. Since the design forces a correlation (*r* = -0.36, see Table D in S1 File for more details) between being foreign-born and Swedish citizenship (i.e., all natives are Swedish citizens), we move on to multivariate regression analysis. To ease the interpretation of our results, we consistently present estimates that rely on a linear probability model. Results using a logistic model are in the S1 File (Table A, Table B and Table C), and are substantively similar unless otherwise noted.

We present two sets of results: one where we pool the applicants born outside of Sweden and one where we do not. The most basic specification (Table 2, column 1) shows that citizenship has a positive, but very small and imprecisely estimated effect (*p*≈0.36), on the callback rate. The results are similar in column 3 (*p*≈0.39). These results do not lend support for the hypothesis that employers are more likely to hire immigrants who have adopted Swedish citizenship.

**Table 2:** CV experiment results. DV: Employer Callback.

	(1)	(2)	(3)	(4)
*Country of birth*:				
Outside Sweden	-0.089*** (0.030)	-0.087*** (0.030)		
Poland			-0.026 (0.034)	-0.024 (0.034)
Iraq			-0.096*** (0.033)	-0.094*** (0.032)
Somalia			-0.146*** (0.031)	-0.144*** (0.031)
Citizenship (Yes)	0.016 (0.017)	0.016 (0.017)	0.014 (0.017)	0.014 (0.017)
Gender (Female)		0.080*** (0.016)		0.079*** (0.016)
Religious Activity (Yes)		-0.005 (0.016)		-0.005 (0.016)
Previous Experience (More)		0.029 (0.019)		0.026 (0.019)
Job requirement (Waiting experience)		0.038 (0.025)		0.043* (0.024)
Observations	1,492	1,492	1,492	1,492
R-squared	0.053	0.075	0.072	0.094
City Dummies	Yes	Yes	Yes	Yes
Time-trend	Yes	Yes	Yes	Yes
Adj. R-squared	0.047	0.067	0.065	0.085

Notes: The dependent variable is a dummy indicating whether applicants were called to an interview (1) or not (0). OLS coefficients; robust standard errors in parentheses. Significance levels:

*** p<0.01,

** p<0.05,

* p<0.1

In columns 2 and 4 of Table 2 we add further treatment conditions. Controlling for all covariates not only increases statistical power, but also allows us to compare the magnitude of the effect of country of birth to that of other characteristics. The point estimates for the effects of country of birth in column 2 closely track those in column 1, suggesting that randomization succeeded. Further, ethnic penalties for Iraqi and Somali applicants are large compared to other treatment conditions.

If it is not obvious that immigrants can improve their labor market chances by adopting citizenship, does the accumulation of work experience help? We find that prior relevant work experience raises the probability of receiving a callback by about 3 percentage points. The effect is thus fairly small and fails to reach conventional levels of statistical significance (*p*>0.1). The callback rates for jobs that require previous cashier and waiting experience are 4 percentage-points higher when compared to jobs that lack this requirement. Finally, explicit mentions of religious activity do not appear to harm applicants.

Summing up, ascriptive traits such as country of birth and gender are much more consequential at the recruitment stage than are characteristics over which immigrants and policymakers have some control, such as citizenship acquisition, work experience, or signals of religious activity. The effects of malleable traits are estimated with a high degree of uncertainty, especially when contrasted with the clear effects of ascriptive traits.

However, it is possible that the effects of some of these variables vary across groups. To test for this possibility, we leverage the factorial design, which allows us to examine whether ethnic hierarchies persist when country of birth is interacted with several important background characteristics. As mentioned earlier, statistical power will be lower when studying interaction (vs. main) effects [16]. This is especially true when we consider factors with several levels, such as country of birth. We therefore report one set of interaction results in Table 3, where we increase statistical power by pooling Polish-, Iraqi- and Somali-born applicants into one group, and one set of more disaggregated results in Table 4. While the disaggregated results come at the price of wider confidence intervals, they are included because they provide a more detailed picture of whether effects vary with applicants’ countries of birth.

**Table 3:** Heterogeneous effects of being born outside of Sweden. DV: Employer Callback.

	(1)	(2)	(3)	(4)
Born outside Sweden (Yes)	-0.087*** (0.030)	-0.071* (0.040)	-0.144*** (0.043)	-0.114*** (0.040)
Citizenship (Yes)		0.016 (0.017)	0.016 (0.017)	0.016 (0.017)
Gender (Female)	0.080*** (0.016)	0.079*** (0.016)	0.081*** (0.016)	0.032 (0.055)
Religious Activity (Yes)	-0.005 (0.016)	-0.006 (0.016)	-0.105* (0.054)	-0.005 (0.017)
Previous Experience (Yes)	0.029 (0.019)	0.010 (0.059)	0.029 (0.019)	0.029 (0.019)
Job requirement (Waiting Experience)	0.038 (0.025)	0.123* (0.074)	0.040 (0.024)	0.039 (0.025)
Born outside Sweden×Citizenship	0.016 (0.017)			
Born outside Sweden×Previous Experience		0.023 (0.063)		
Born outside Sweden×Job Requirement		-0.101 (0.078)		
Born outside Sweden×Religious Activity			0.117** (0.057)	
Born outside Sweden×Gender(Female)				0.056 (0.057)
Observations	1,492	1,492	1,492	1,492
R-squared	0.075	0.077	0.079	0.076
City Dummies	Yes	Yes	Yes	Yes
Time-trend	Yes	Yes	Yes	Yes

Notes: The dependent variable is a dummy indicating whether applicants were called to an interview (1) or not (0). OLS coefficients; robust standard errors in parentheses. Significance levels:

*** p<0.01,

** p<0.05,

* p<0.1

**Table 4:** Heterogeneous effects of country of birth. DV: Employer Callback.

	(1)	(2)	(3)	(4)
*Country of birth*:				
Poland	-0.031 (0.037)	-0.018 (0.045)	-0.086* (0.048)	-0.079* (0.044)
Iraq	-0.116** (0.048)	-0.082* (0.042)	-0.157*** (0.047)	-0.114*** (0.042)
Somalia	-0.154*** (0.047)	-0.114*** (0.041)	-0.192*** (0.044)	-0.152*** (0.040)
Citizenship (Yes)		0.014 (0.017)	0.014 (0.017)	0.015 (0.017)
Gender (Female)	0.079*** (0.016)	0.078*** (0.016)	0.079*** (0.016)	0.032 (0.055)
Religious Aciticity (Yes)	-0.005 (0.016)	-0.006 (0.016)	-0.105* (0.054)	-0.005 (0.016)
Previous Experience (Yes)	0.026 (0.019)	0.010 (0.059)	0.026 (0.019)	0.027 (0.019)
Job requirement (Waiting Experience)	0.043* (0.024)	0.123* (0.074)	0.045* (0.024)	0.044* (0.024)
*Country of birth*×*Citizenship*:				
Poland×Citizenship	0.001 (0.035)			
Iraq×Citizenship	0.032 (0.045)			
Somalia×Citizenship	0.007 (0.041)			
*Country of birth*×*Previous Experience*:				
Poland×Previous Experience		0.032 (0.071)		
Iraq×Previous Experience		0.032 (0.068)		
Somalia×Previous Experience		-0.007 (0.064)		
*Country of birth*×*Job Requirement*:				
Poland×Job Requirement		-0.084 (0.091)		
Iraq×Job Requirement		-0.101 (0.085)		
Somalia×Job Requirement		-0.099 (0.081)		
*Country of birth*×*Religious Activity*:				
Poland×Religious Activity			0.125* (0.065)	
Iraq×Religious Activity			0.128** (0.061)	
Somalia×Religious Activity			0.097* (0.058)	
*Country of birth*×*Gender (Female)*:				
Poland×Gender(Female)				0.109* (0.065)
Iraq×Gender(Female)				0.042 (0.062)
Somalia×Gender(Female)				0.01 (0.059)
Observations	1,492	1,492	1,492	1,492
R-squared	0.094	0.097	0.098	0.098
City Dummies	Yes	Yes	Yes	Yes
Time-trend	Yes	Yes	Yes	Yes
Adj. R-squared	0.084	0.084	0.087	0.087

Notes: The dependent variable is a dummy indicating whether applicants were called to an interview (1) or not (0). OLS coefficients; robust standard errors in parentheses. Significance levels:

*** p<0.01,

** p<0.05,

* p<0.1

Tables 3 and 4 interact citizenship with countries of birth (recall that citizenship is held constant for the Swedish-born). The citizenship effect varies slightly across groups, but none of these interactions reach conventional levels of statistical significance. For example, the 95% confidence interval around the estimated effect of citizenship in Table 3 ranges from a small negative effect of -2 percentage points to a moderately-sized positive effect of 4 percentage points. (In Table E in the S1 File we address concerns related to multiple comparisons by presenting results controlling for the false discovery rate [30].)

The second columns of Tables 3 and 4 presents the impact of being foreign-born conditional on previous experience. If statistical discrimination related to uncertainty about relevant background experience is at work, employers who receive CVs that contain information about such experience should discriminate less. The interactions in question are, however, substantively small and imprecisely estimated.

Turning to an additional aspect of experience, in our design we distinguished between two types of jobs, those that require previous waiting experience and those that do not. The former always receive applications indicating more previous experience whereas the latter receive applications of both types. This design forces a correlation (*r* = .53) between previous experience and whether the job requires prior waiting experience, and therefore the interaction between country of birth and job requirement is also included in column 2 of Table 3 as well as of Table 4. The interactions between job type and country of birth are quite large and negative, potentially suggesting that employers are reluctant to hire immigrants for positions which, on average, are likely to entail more customer contact. However, though large in magnitude, these interaction effects fall below conventional levels of statistical significance.

We also examine whether employers react to signals of religiosity. Research has shown that employers in France discriminate against Muslim applicants, even when country of birth and other characteristics are held constant [10]. Our design does not allow us to tease apart religion, country of birth, and ethnicity: the great majority of Iraqi and Somali migrants are Muslim, and, to maintain realism and external validity, we did not vary their denomination. We instead chose to include signals of religiosity. That is, holding presumed denomination within a given country of birth constant, we assess whether priming religiosity influences employer behavior (for a similar approach, see [31–33]). We do so because some of the concerns about employing Muslims could relate to the group’s religious needs (e.g., the frequency of prayer or the provision of halal foods) rather than their denomination per se, and how these needs interfere with productivity or company policies. Accordingly, if employers receive information that Muslim applicants indeed practice their religion, this could depress callback rates.

To test this hypothesis, half of the Somali- and Iraqi-born applicants indicated that they were active in a local mosque. If bias against religiously-practicing Muslims were driving the results, we would expect Iraqis and Somalis to fare even worse when they indicate their religious involvement. Column three of Table 4 demonstrates that this is not the case. If anything, the ethnic penalty for religiously active Iraqi- and Somali-born applicants is smaller compared to that among the non-active compatriots, as can be seen from the interaction between the signal of religious activity and country of birth. For instance, Somali-born applicants that do not signal religious activity have a 19 percentage points lower callback rate than Swedish natives, whereas the penalty for those who do is almost 10 percentage points smaller. This provides suggestive evidence that discrimination against Iraqi- and Somali-born jobseekers is not solely driven by their degree of religiosity and the practical aspects related to accommodating religious Muslims at the workplace. Moreover, among Polish-born applicants, those who are religiously active (i.e., involved in a local Catholic Church), also face a smaller ethnic penalty. For all foreign-born groups, then, ethic penalties are smaller when they signal religious activity (see also column 3 of Table 3). By contrast, indicating participation in a local Protestant church does reduce callbacks among native Swedes (*p* < 0.10).

In sum, the evidence does not support the conclusion that signals of religious practice (vs. denomination per se) among the foreign-born compound discrimination. Instead, the differences in the effects of religious activity across natives and immigrants are more in line with the role congruity theory of prejudice [34], in that candidates born in the largely secular, but historically Protestant, Sweden appear to be evaluated less favorably when practicing their religion, whereas candidates born in more religious parts of the world do not face an additional penalty when they make explicit mention of practicing the dominant religion of their country of birth.

We next turn to interactions of country of birth and gender (column 4). The estimates in column four of Tables 3 and 4 suggest that ethnic penalties are smaller for women than for men. The interaction between country of birth and gender is, however, only statistically significant (*p*<.10) for the Polish-born.

**Fig 3:**
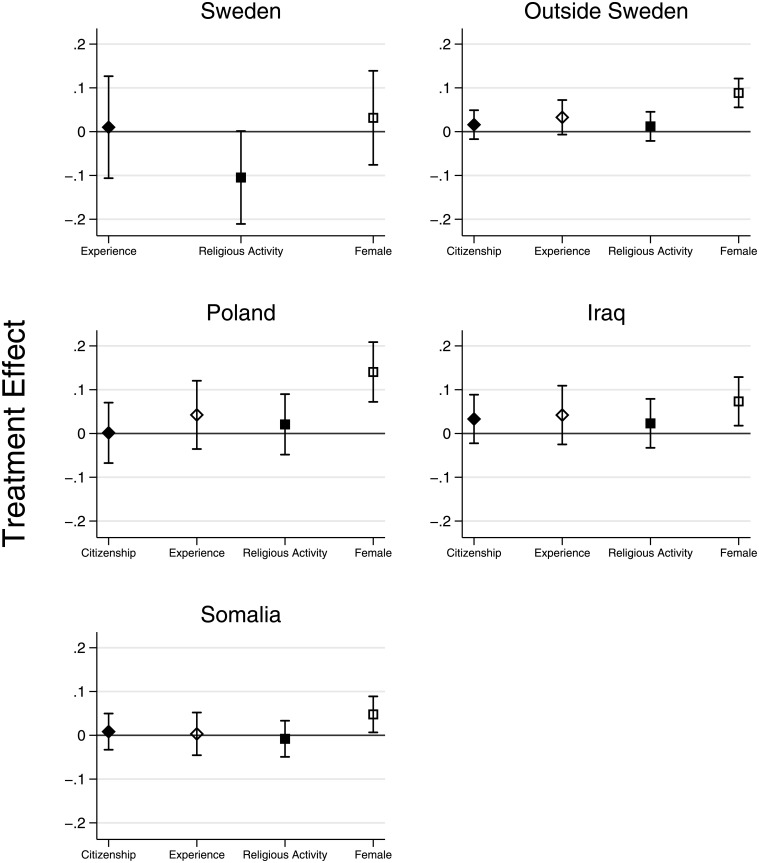
Impact of treatments on callback rates by country of birth. Note: Capped bars represent 95% confidence intervals.

Finally, do attributes that immigrants can affect—the adoption of citizenship, the accumulation of skills, and signals of religious practice—influence recruitment prospects? Fig 3 presents the marginal effects of all other treatments conditional on country of birth (based on Tables 3 and 4). Only gender matters consistently: Being a woman has a positive and statistically significant effect among all immigrant groups (For details on subgroup comparisons, see Appendix C in the S1 File).

These findings conflict with accounts that document the multiple burdens of being a woman and belonging to a minority ethnic group [35]. Keeping in mind that we are considering migrants who have attained some degree of integration (recall the baseline attractiveness communicated in the cover letter and the potential football-related gender effects among Muslims), they are more consistent with role theories postulating that stereotypes about foreign nationalities are mainly ascribed to males [36]. Notably, among Swedish-born applicants there is little penalty for being male, suggesting that the gendered nature of callback rates among immigrants is not driven by a general, industry-specific preference for female labor.

## Discussion

Using a factorial field experiment, our study has gone beyond most existing work in simultaneously testing the impact of a number of factors on discrimination in the hiring process. Our results paint a bleak picture: We find substantial evidence in support of employer discrimination against immigrants and no consistent evidence that immigrants can reduce this discrimination by acquiring citizenship or investing in job-related experience. Ethnic hierarchies decisively influence immigrants’ employment prospects. Though employers discriminate against all immigrant groups, callback rates decline significantly with the degree of socio-cultural and ethnic distance, leaving Iraqis and especially Somalis with much reduced employment chances. Moreover, among these groups, men fare particularly poorly. It bears repeating that Somali men only receive callbacks to 2.5 percent of applications, while the comparable number is 19 percent for Swedish men. Compared to these ascriptive traits, the effects of malleable traits are much smaller, and the confidence intervals surrounding these effects are wide.

These outcomes are disconcerting on normative and practical grounds. Employers subscribe to an ethnic hierarchy when judging applicants, and their assessments are seemingly impervious to attributes that signal productivity or integration. Fixed traits such as country of birth and gender over which applicants have no control are given much more weight in the hiring process than are characteristics that immigrants and policy measures can actually affect.

Turning to policy implications, the fact that immigrants confront severe obstacles even in low-skill entry-level jobs, and that they cannot easily address these obstacles on their own, calls for policy interventions that minimize the extent and impact of employer prejudice.

## Materials and methods

### Ethics statement

This research involves human subjects. Consent from participants was not obtained as that would undermine study credibility. The experimental data set has been anonymized by Vernby, who carried out the data analysis. While no individual names were saved, a data set that contains the names of the included establishments and their company registration number is kept by Vernby in a separate encrypted file on a password protected server. The research was conducted in Sweden and was approved by the Regional Ethical Review Board in Stockholm on September 3, 2015 (see approval number 2015/1352-31/5).

### Empirical strategy for the registry data analysis

We use anonymized registry data based on administrative records and collected by the government statistical agency Statistics Sweden. These data cover the entire Swedish population, and we restrict our analysis to members of the working-age population (ages 15-64).

For reasons of confidentiality, the “county-of-birth” variable aggregates countries that have sent relatively small numbers of migrants to Sweden into a regional category. This applies to Somalia, which is grouped with Eritrea, Ethiopia, Sudan and Djibouti. This grouping should not compromise our ability to document ethnic hierarchies. In 2015, the Somali-born made up more than 55% of this group (the second largest sender, Eritrea, made up 26%). Moreover, like Somalia, Djibouti and Sudan are majority Muslim, whereas Muslims constitute a significant (but not a majority) share of the population in Eritrea and Ethiopia. However, our ability to document ethnic hierarchies could be slightly confounded by this aggregation if migrants from Eritrea are more economically integrated than are those from Somalia. Fortunately, the CV study does not face similar methodological problems.

The Swedish citizenship measure relies on a variable that indicates the date on which an individual has changed citizenship, but it does not indicate an individuals’ nationality.

### Design of the CV study

#### Experimental design

The experiment employs a fractional factorial design. The advantage of this design is that it allows us to study the main effects of several factors using the same sample size that would be required for the study of one independent variable without loss of statistical power. Moreover, the factorial design allows testing for interactions between treatments. Note, however, that when studying interaction effects statistical power will be lower, resulting in wider confidence intervals than for main effects [16]. Additionally, if the combination of factors studied are both unrepresentative of real-wold settings, and exhibit strong interaction effects, this can limit the external validity of treatment effects from factorial experiments [37].

We randomly vary gender (Male/Female), post high-school work-experience (Yes/No), religious activity (Yes/No), country of birth (Sweden, Poland, Somalia, Iraq) and, for the foreign-born, Swedish citizenship (Yes/No). The job openings that were coded as requiring cashier and waiting staff experience only received applications with post high-school work-experience, whereas the ones that listed no such requirement received CVs both with and without post high-school work experience.

#### Occupational sector

To ensure maximum relevancy we chose to focus on the occupational sector in which the highest proportion of immigrants is employed. If we uncover discrimination in such a sector, we can conclude that discrimination has important consequences for the employment prospects of immigrants and, moreover, that our estimates of discrimination potentially represent a lower bound. In the Swedish case, this sector is the restaurant sector. Furthermore, given the focus on young adults (applicants are 21 to 22 years old) and the high level of youth unemployment in Sweden, jobs in the restaurant sector are suitable because they frequently represent entry-level occupations.

#### Geographical area

Our study focuses on the labor markets in the biggest city regions: Stockholm, Malmö, Gothenburg, Örebro, Västerås, Uppsala, Norrköping and Linköping. Again, this choice was made to ensure relevance. About 3 million of Sweden’s 10 million inhabitants live in these regions. In addition, immigrants disproportionately live in cities, so if discrimination occurs in the biggest city regions this has important consequences for the employment prospects of immigrants.

#### Time frame

The experiment started in February 2016 and ended in May 2017.

#### Further selection criteria

Applications were sent by email to all job ads that fit the above requirements and that were announced on the Swedish Public Employment Agency’s website Platsbanken. Ads that required applicants to fill out a pre-determined form had to be excluded since there was no way to follow the experimental protocol in such cases. We sent only one (randomly selected) application to each job opening because, first, we were concerned that sending several CVs to the same job opening would increase the risk of detection and, second, we worried about ethical implications. Specifically, we wanted to minimize the time spent by employers on fictitious applicants and to avoid the risk that employers would overestimate the labor supply, which could in turn affect their hiring and treatment of future employees.

#### Coding of responses

Of the 56 possible CVs, we randomly selected one to be sent to each opening. We registered replies in two ways. Positive responses (coded 1) most often came in the form of an invitation to interview, but they could also consist of requests for more information (e.g., “Are you still interested in working for us?”) or direct offers of work. All other responses and non-responses were coded 0.

#### Construction of CVs

The names were chosen based on common names in the fictitious applicant’s country of birth. To test the robustness of our results to the specific choice of name we used two female and two male names for each country of birth (see Appendix A in the S1 File). Treatment effects did not differ at conventional levels of statistical significance for any of the eight pairs of names (results are available from the authors). For people born in Poland, Iraq and Somalia, the city of birth was the capital of the country (Warsaw, Baghdad and Mogadishu, respectively). For people born in Sweden, the city of birth was one of the following (depending on which city the job opening was located in): Stockholm, Malmö, Gothenburg, Örebro, Västerås, Uppsala or Linköping. The previous residential area where the applicant went to primary and upper secondary school (the latter with a social science program) and current address were chosen with regard to average income and demographics, and we selected an existing public upper secondary school (with a social science program) in the previous residential area. The resumé contained no description of a specific primary school but was described as follows: Degree primary school, [previous residential area]. All persons (including the Swedish-born applicants) were described as having Swedish, English and some German language skills. Applicants who were born abroad indicated an additional language based on their country of birth.

Three fictitious workplaces were used in the CVs. All applicants had worked at a café during upper secondary school. Their resumés also stated an internship at a cinema. Half of the applicants had additional work experience as a waiter at a restaurant since high school (these are considered as having more experience). The CV also included associational memberships. It stated that the applicant was member of football club in his/her residential area, and the club was therefore selected separately for each city. The choice of football is motivated by the fact that it is the most commonly practiced team-sport among the young according to *The Swedish Sports Confederation*. In particular, 26% of those between 15 and 24 played, and while there is a gender imbalance (34% of the men and 16% of the women in this age group played), it is the most common team sport among both men and women in this age category. The candidate’s interest in football also provided a vehicle to mention the positive qualities he/she had acquired due to her engagement in team sports.

Additionally, half of the CVs included membership in a religious congregation. Like in many previous correspondence studies on the impact of religious affiliation on hiring discrimination, we signal religious affiliation by having applicants report activities in religious groups. We selected the denomination and associated congregation on the basis of the dominant religion in the country of birth. The religious congregation was selected separately for each city. In the S1 File (Fig A and Fig B) we present translated CVs of a male Iraqi-born Swedish citizen who is active in a mosque in Stockholm (with more or less previous work experience, respectively).

#### Cover letters

All cover letters began with a self-description of the applicant as cooperative, ambitious and resourceful followed by a description of his or her commitment to football, with the focal point on the positive qualities which this commitment generated. While football was chosen because it is popular both among young men and women, as well as among natives and immigrants, it could arguably be a stronger signal of integration for Muslim women. To the extent that this is true, ethnic penalties could be slightly underestimated for female Muslim job candidates. Nonetheless, we find ethnic penalties as large as 14 percentage points for Somali women (See Table 4). The mention of football was followed by a discussion of previous work experience. To increase the response rate, the work experience matched the qualities that were commonly mentioned in work ads. For example, resumés with experience from before graduation at a café stated cashier and barista skills. Resumés with work experience gained after graduation at a restaurant stated skills such as table service and management. Fig C and Fig D in the S1 File present translated cover letters of a male Iraqi-born Swedish citizen who is active in a mosque in Stockholm (with more or less previous work experience, respectively).

#### Pilot study

Between January and February in 2016, we carried out a pilot study in the Stockholm labor market. We sent applications to 90 job openings announced on the Swedish Public Employment Agency’s website Platsbanken. Since the callback rate was low (5 invitations), we implemented a number of changes to make our fictitious candidates more attractive to employers. First, the format of the CVs and the cover letters should be fairly typical of young applicants, as we drafted them based on advice from the Swedish Public Employment Agency. However, due to the low callback rate, we improved the layout of the CVs. Second, we enhanced the cover letters and CVs, adding more detailed information about skills that are generally relevant to working in the category of jobs studied. These two changes were implemented in an identical fashion in all cover letters and CVs and thus likely raised the general quality of all our candidates. Third, CVs in the pilot study varied whether applicants had relevant work experience during high school. To increase the response rate in the main study, we decided that all applications would include this experience (and we varied post high-school work experience). This design choice potentially makes our applicants somewhat more attractive than the average real-world applicant.

## Supporting information

S1 File Additional information, tables and figures. (PDF)
