# Supplementary material for: Can immigrants counteract employer discrimination? A factorial field experiment reveals the immutability of ethnic hierarchies
Source: PLoS One. 2019 Jul 24;14(7):e0218044. doi: 10.1371/journal.pone.0218044 (PMC6655538; doi:10.1371/journal.pone.0218044)
Supplement: S1 File — (PDF) [file pone.0218044.s001.pdf]

Supporting information

Appendix A. Additional information on the construction of CVs.
Swedish names: Malin Svensson, Emma Lindberg, Erik Svensson and Filip Lindberg. Polish names: Julia Kowalski, Maria Nowak, Antoni Kowalski and Mateusz Nowak. Iraqi names: Sarah Amari, Zainab Hadad, Ahmed Amari and Ali Hadad. Somali names: Awa Zakaria, Fatima Mohamed, Khalid Zakaria and Abdi Mohamed. Treatment effects did not differ at conventional levels of statistical significance for any of the eight pairs of names.

Appendix B. Overview of correspondence studies of ethnic discrimination since 2005
Baert [1] has created a register of all correspondence studies since Bertrand and Mullainathan [2]. We have reviewed those studies in the register that test for ethnic discrimination and asked: (1) Do they have a design that allows for the testing of ethnic hierarchies and (2) How many experimental factors do they include? Most of the studies (70%) do not have a design that allows for the testing of ethnic hierarchies and the average number of experimental factors tested is around 2 (typically ethnicity and gender). Of the studies in which more than two ethnicities are tested, there are only three that study more than 2 experimental factors. *S6 Table* summarizes the reviewed studies.

The one existing study that, from a design-perspective, resembles our study is by Arceo-Gomez and Campos-Vazques [3], which studies callback rates in the Mexican labor market for whites, mestizos and indigenous candidates. Apart from ethnicity, the authors study three other factors: gender (male/female), if the candidate is married (yes/no) and if he/she went to a private or public university. While the study resembles ours in that it has a design that allows for the testing of ethnic hierarchies, and in that it varies a larger number of experimental factors than the typical correspondence study, it is carried out in a very different context, and does not focus on discrimination of immigrants from different parts of the world. Moreover, it does not test whether signals of integration, such as work experience and citizenship, can reduce discrimination.

Appendix C. Which subgroup comparisons are significantly different from the others in Fig 4?
When comparing effects sizes of our treatments across different countries of birth, we find that the impact of religious activity is significantly higher for Polish-born than for Swedish-born ($p < 0.10$), Iraqi-born than for Swedish-born ($p < 0.05$) and Somali-born than for Swedish-born ($p < 0.10$). In addition, the impact of being female is higher for Polish-born than for Swedish-born ($p < 0.10$) and for Polish-born than for Somali-born ($p < 0.05$).

Fig. A. Example of a CV with less previous experience.

Ahmed Amari

Prästgårdsgatan 45, 172 32 Sundbyberg, Stockholm | Telephone:
0739691921 | ahmed.amari776@gmail.com

About me

I was born in 1995 in Baghdad, but came to sweden as a 6-year-old and became a swedish citizen two years ago. I am at the moment living in Sundbyberg. In my spare time I like to spend time with my family and friends. Football is also a great interest of mine. Through it, I have developed my ability to work with diffrent types of people, which allows me to easily adapt to new contexts. I'm sure i will fit in well in your workplace.

Education

- 2011-2014 Social Science Programme, Solna Upper secondary school, Solna
- 2011 Degree Primary school, Solna

Work experience

RESTAURANT ASSISTANT/BARISTA | KANELBULLENS CAFÉ | 2011-2014

- The tasks included making simple meals and drinks, cash assistent, dishwashing and cleaning.

INTERNSHIP | KANELBULLENS CAFÉ | 2011

- The tasks included cleaning and making simple meals and drinks.

INTERNSHIP | SF FILMSTADEN KISTA | 2010

- The tasks included cleaning of movie theaters and ticketing.

Other associations

SPÅNGA IS FOTBOLL

STOCKHOLMS MOSKÉ

Other qualifications

DRIVER'S LICENCE

LANGUAGES:

- English, swedish, arabic and some german

Fig. B. Example of a CV with more previous experience

Ahmed Amari

Prästgårdsgatan 45, 172 32 Sundbyberg, Stockholm | Telephone: 0739691921 | ahmed.amari776@gmail.com

About me

I was born in 1995 in Baghdad, but came to sweden as a 6-year-old and became a swedish citizen two years ago. I am at the moment living in Sundbyberg. In my spare time I like to spend time with my family and friends. Football is also a great interest of mine. Through it, I have developed my ability to work with diffrent types of people, which allows me to easily adapt to new contexts. I'm sure i will fit in well in your workplace.

Education

- 2011-2014 Social Science Programme, Solna Upper secondary school, Solna
- 2011 Degree Primary school, Solna

Work experience

WAITER | BISTRO LA MÉDITERRINÉE | 2014-

- The tasks included table service and management of the restaurant's reservations.

RESTAURANT ASSISTANT/BARISTA | KANELBULLENS CAFÉ | 2011-2014

- The tasks included making simple meals and drinks, cash assistent, dishwashing and cleaning.

INTERNSHIP | KANELBULLENS CAFÉ | 2011

- The tasks included cleaning and making simple meals and drinks.

INTERNSHIP | SF FILMSTADEN KISTA | 2010

- The tasks included cleaning of movie theaters and ticketing.

Other associations

SPÅNGA IS FOTBOLL

STOCKHOLMS MOSKÉ

Other qualifications

DRIVER'S LICENCE

LANGUAGES:

- English, swedish, arabic and some german

Fig. C. Example of a cover letter with less previous experience

Ahmed Amari

Prästgårdsgatan 45, 172 32 Sundbyberg, Stockholm | Telephone:
0739691921 | ahmed.amari776@gmail.com

Cover letter

I quickly realized after finding your work ad at Platsbanken that this job suits me perfectly.

I am a cooperative, ambitious and resourceful person. People around me perceive me as a very positive and energetic person. I have a great interest in sport, especially football. Through my involvment in football, I have developed a great ability to cooperate, which i think will be a great advantage in this type of work. It is also by experience in team sports I have developed my ambitious side. I have a habit of always striving for the best possible results and my ability to take initiatives is often an advatage in these situations.

With my previous work experience, i believe I have a good foundation to stand on. Through my experience of working as a restaurant assistant at Kanelbullens café, I have developed my social skills that can come in handy in many situations. I have also developed the ability to adapt to diffrent situations that may occur. I also have good skills as a cashier and basic barista skills. I like working with people and always strive to provide a good service.

I am applying for this job because I am searching for new challenges. I am convinced that I will learn a lot but also contribute much. I will tell you more about myself during a possible interview!

Greetings,

Ahmed

Fig. D. Example of a cover letter with more previous experience

Ahmed Amari

Prästgårdsgatan 45, 172 32 Sundbyberg, Stockholm | Telephone:
0739691921 | ahmed.amari776@gmail.com

Cover letter

I quickly realized after finding your work ad at platsbanken that this job suits me perfectly.

I am a cooperative, ambitious and resourceful person. People around me perceive me as a very positive and energetic person. I have a great interest in sport, especially football. Through my involvment in football, I have developed a great ability to cooperate, which I think will be a great advantage in this type of work. It is also by experience in team sports I have developed my ambitious side. I have a habit of always striveing for the best possible results and my ability to take initiatives is often an advatage in these situations.

With my previous work experience, I believe I have a good foundation to stand on. Through my experience of working as a restaurant assistant at Kanelbullens café, I have developed my social skills that can come in handy in many situations. I have also developed the ability to adapt to diffrent situations that may occur. I also have good skills as a cashier and basic barista skills.

I like working with people and always strive to provide a good service. In my previous work as a waiter at Bistro la Méditerrinée, I have had the opportunity to further develop my social skills and also learn to handle stressful situations. I am also very familiar with the skills required to be a waiter, for example serving dishes at tables according to order and cash handling and credit card payment procedures.

I am applying for this job because I am searching for new challenges. I am convinced that I will learn a lot but also contribute much. I will tell you more about myself during a possible interview!

Greetings,

Ahmed

Table A. CV Experiment Results. DV: Employer Callback. Logit estimates.

	(1)	(2)	(3)	(4)
Country of birth:				
Outside Sweden	-0.690***	-0.694***		
	(0.219)	(0.222)		
Poland			-0.142	-0.126
			(0.238)	(0.242)
Iraq			-0.763***	-0.766***
			(0.262)	(0.268)
Somalia			-1.549***	-1.578***
			(0.302)	(0.304)
Citizenship (Yes)	0.194	0.205	0.184	0.195
	(0.186)	(0.188)	(0.189)	(0.191)
Gender (Female)		0.837**		0.852***
		(0.175)		(0.178)
Religious Activity (Yes)		-0.030		-0.031
		(0.168)		(0.170)
Previous Experience (More)		0.339*		0.345*
		(0.202)		(0.203)
Job requirement (Waiting experience)		0.277		0.315
		(0.217)		(0.219)
Observations	1,492	1,492	1,492	1,492
Pseudo-R-squared	0.067	0.098	0.097	0.130
City Dummies	Yes	Yes	Yes	Yes
Time-trend	Yes	Yes	Yes	Yes

Notes: The dependent variable is a dummy indicating whether applicants were called to an interview (1) or not (0). Logit coefficients; robust standard errors in parentheses. Significance levels: *** p<0.01, ** p<0.05, * p<0.1

Table B. Heterogeneous effects of being born in or outside of Sweden. DV: Employer Callback. Logit estimates.

	(1)	(2)	(3)	(4)
Born outside Sweden (Yes)	-0.694***	-0.667**	-1.114***	-1.172***
	(0.222)	(0.336)	(0.292)	(0.323)
Citizenship (Yes)		0.209	0.201	0.206
		(0.188)	(0.188)	(0.189)
Gender (Female)	0.837***	0.827***	0.849***	.223
	(0.175)	(0.175)	(0.176)	(0.361
Religious Activity (Yes)	-0.030	-0.039	-0.722*	-0.020
	(0.168)	(0.168)	(0.374)	(0.169)
Previous Experience (Yes)	0.339*	0.159	0.330	0.332
	(0.202)	(0.446)	(0.202)	(0.203)
Job requirement (Waiting Experience)	0.277	0.632	0.307	0.305
	(0.217)	(0.454)	(0.217)	(0.219)
Born outside Sweden×Citizenship	0.205			
	(0.188)			
Born outside Sweden×Previous Experience		0.225		
		(0.499)		
Born outside Sweden×Job Requirement		-0.459		
		(0.514)		
Born outside Sweden×Religious Activity			0.889**	
			(0.422)	
Born outside Sweden×Gender(Female)				0.810*
				(0.416)
Observations	1,492	1,492	1,492	1,492
Pseudo R-squared	0.098	0.099	0.102	0.102
City Dummies	Yes	Yes	Yes	Yes
Time-trend	Yes	Yes	Yes	Yes

Notes: The dependent variable is a dummy indicating whether applicants were called to an interview (1) or not (0). OLS coefficients; robust standard errors in parentheses. Significance levels: *** p<0.01, ** p<0.05, * p<0.1

Table C. Heterogeneous effects of country of birth. DV: Employer Callback. Logit estimates.

	(1)	(2)	(3)	(4)
Country of birth:				
Poland	-0.031	-0.098	-0.562*	-0.665*
	(0.528)	(0.367)	(0.323)	(0.365)
Iraq	-0.863	-0.832**	-1.259***	-1.137***
	(0.554)	(0.404)	(0.378)	(0.404)
Somalia	-1.568***	-1.489***	-1.861***	-2.213***
	(0.355)	(0.469)	(0.414)	(0.538)
Citizenship (Yes)		0.200	0.191	0.198
		(0.191)	(0.192)	(0.193)
Gender (Female)	0.850***	0.845***	0.868***	0.215
	(0.178)	(0.178)	(0.180)	(0.362)
Religious Aciticity (Yes)	-0.034	-0.038	-0.727*	-0.020
	(0.170)	(0.171)	(0.376)	(0.171)
Previous Experience (Yes)	0.344*	0.164	0.338*	0.337*
	(0.203)	(0.447)	(0.202)	(0.204)
Job requirement (Waiting Experience)	0.319	0.626	0.339	0.352
	(0.219)	(0.455)	(0.220)	(0.221)
Country of birth× Citizenship:				
Poland×Citizenship	0.049			
	(0.267)			
Iraq×Citizenship	0.410			
	(0.340)			
Somalia×Citizenship	0.215			
	(0.459)			
Country of birth× Previous Experience:				
Poland×Previous Experience		0.193		
		(0.543)		
Iraq×Previous Experience		0.388		
		(0.600)		
Somalia×Previous Experience		0.041		
		(0.718)		
Country of birth× Job Requirement:				
Poland×Job Requirement		-0.420		
		(0.575)		
Iraq×Job Requirement		-0.455		
		(0.619)		
Somalia×Job Requirement		-0.297		
		(0.744)		
Country of birth× Religious Activity:				
Poland×Religious Activity			0.922**	
			(0.464)	
Iraq×Religious Activity			1.017**	
			(0.512)	
Somalia×Religious Activity			0.620	
			(0.594)	
Country of birth× Gender (Female):				
Poland×Gender(Female)				0.916*
				(0.469)
Iraq×Gender(Female)				0.654
				(0.062)
Somalia×Gender(Female)				1.024
				(0.650)
Observations	1,492	1,492	1,492	1,492
Pseudo-R-squared	0.131	0.135	0.134	0.131
City Dummies	Yes	Yes	Yes	Yes
Time-trend	Yes	Yes	Yes	Yes

Notes: The dependent variable is a dummy indicating whether applicants were called to an interview (1) or not (0). Logit coefficients; robust standard errors in parentheses. Significance levels: *** p<0.01, ** p<0.05, * p<0.1

Table D. Correlations between treatments.

	Citizenship (Yes)	Religious Acitivity (Yes)	Previous Experience (Yes)	Sweden	Poland	Iraq	Somalia	Gender (Female)
Religious Acitivity (Yes)	-0.01							
	(0.69)							
Previous Experience (Yes)	-0.00	-0.00						
	(0.95)	(0.99)						
Sweden	0.36	-0.02	0.02					
	(0.00)	(0.55)	(0.41)					
Poland	-0.08	0.00	0.01	-0.26				
	(0.00)	(0.95)	(0.68)	(0.00)				
Iraq	-0.10	0.02	-0.03	-0.26	-0.40			
	(0.00)	(0.49)	(0.31)	(0.00)	(0.00)			
Somalia	-0.10	-0.01	-0.00	-0.26	-0.40	-0.40		
	(0.00)	(0.78)	(0.97)	(0.00)	(0.00)	(0.00)		
Gender (Female)	-0.01	-0.02	-0.02	-0.01	0.01	-0.01	-0.00	
	(0.70)	(0.50)	(0.50)	(0.83)	(0.59)	(0.83)	(0.88)	
Job requirement	0.04	-0.04	0.53	0.03	-0.05	0.03	-0.00	0.02
	(0.17)	(0.15)	(0.00)	(0.20)	(0.06)	(0.31)	(0.88)	(0.35)

Notes: Pearson's r. Entries in parentheses are p-values. Shaded areas show treatments that are correlated by design.

Table E. Main treatment effects and differences in treatment effects across countries of origin using Benjamini-Hochberg method to control for false discovery rate.

	Main effects	Sweden-Poland	Sweden-Iraq	Sweden-Somalia	Poland-Iraq	Poland-Somalia	Iraq-Somalia
Poland	-0.024						
	(0.470)						
Iraq	-0.094#						
	(0.004)						
Somalia	-0.144#						
	(0.000)						
Citizenship (Yes)	0.014				-0.032	-0.007	0.025
	(0.390)				(0.484)	(0.864)	(0.484)
Previous Experience (Yes)	0.026	-0.032	-0.032	0.007	0.000	0.039	0.039
	(0.167)	(0.651)	(0.640)	(0.915)	(0.995)	(0.402)	(0.357)
Job Requirement	0.043	0.084	0.101	0.099	0.017	0.015	-0.002
	(0.078)	(0.354)	(0.237)	(0.221)	(0.807)	(0.810)	(0.974)
Religious Activity (Yes)	-0.005	-0.125	-0.128	-0.097	-0.002	0.029	0.031
	(0.759)	(0.052)	(0.037)	(0.095)	(0.959)	(0.483)	(0.378)
Gender (Female)	0.079#	-0.109	-0.042	-0.016	0.067	0.093	0.026
	(0.000)	(0.094)	(0.498)	(0.782)	(0.135)	(0.022)	(0.467)

Notes: Entries are differences in treatment effects based on Table 2 and Table 4 in the main text. Entries in parentheses are p-values. # significant when false discovery rate is set to 0.05.

Table F. Overview of correspondence studies of ethnic discrimination since 2005.

Study	Country	Allows for testing of ethnic hierarchies	Number of experimental factors
Cediey & Foroni (2008)	France	No	1
Edo et al. (2013)	France	No	3
Nunley et al. (2015)	US	No	3
Gaddis (2015)	US	No	5
Jacquement & Yannelis (2012)	US	No	1
Agan & Starr (2016)	US	No	4
Bursell (2014)	Sweden	Yes	2
Darolia et al. (2016)	US	Yes	2
Decker et al. (2015)	US	Yes	2
McGinnity & Lunn (2011)	Ireland	Yes	1
Wood et al. (2009)	UK	Yes	2
Drydakis & Vlassis (2010)	Greece	No	1
Drydakis (2012a)	Greece	No	1
Drydakis (2017)	Greece	No	3
Andriessen et al. (2012)	Netherlands	Yes	2
Derous et al. (2012) TEST1	Netherlands	No	2
Derous et al. (2012) TEST2	Netherlands	No	2
Agerström et al. (2012)	Sweden	No	3
Arai et al. (2015)	Sweden	No	2
Widner & Chicoine (2011)	US	No	1
Bartoš et al. (2014)	Czech Republic	Yes	1
Asali et al. (2017)	Georgia	No	2
Booth et al. (2012)	Australia	Yes	2
Weichselbaumer (in press)	Austria	Yes	2
Baert et al. (2017)	Belgium	Yes	3
Galarza & Yamada (2014)	Peru	No	2
Lee & Khalid (2016)	Malaysia	No	2
Carlsson (2010)	Sweden	No	1
Carlsson & Rooth (2007)	Sweden	No	1
Attström (2007)	Sweden	No	1
Arceo-Gomez & Campos-Vazques (2014)	Mexico	Yes	4
Maurer-Fazio (2013)	China	Yes	3
Pierné (2013)	France	No	2
Berson (2012)	France	No	2
Duguet et al. (2010)	France	No	4
Birkelund et al. (2017)	Norway	No	3
Midtbøen (2016)	Norway	No	2
Baert et al. (2015)	Belgium	No	1
Baert & Vujić (2016)	Belgium	No	3
Kaas & Manger (2012)	Germany	No	2

References

1. Baert S. "An overview of (almost) all correspondenceexperiments since 2005" in SM Gaddis ed. Audit Studies. Berlin: Springer; 20018.

2. Bertrand M, Mullainathan S. Are Emily and Greg more employable than Lakisha and Jamal? A field experiment on labor market discrimination. The American Economic Review 2004; 94(4):991–1013.

3. Arceo-Gomez EO, Campos-Vazquez RM. Race and marriage in the labor market: A discrimination correspondence study in a developing country. The American Economic Review 2014; 104(5):376–380.
